# Insulin resistance genetic risk score and burden of coronary artery disease in patients referred for coronary angiography

**DOI:** 10.1371/journal.pone.0252855

**Published:** 2021-06-18

**Authors:** Regitze Skals, Maria Lukács Krogager, Emil Vincent R. Appel, Theresia M. Schnurr, Christian Theil Have, Gunnar Gislason, Henrik Enghusen Poulsen, Lars Køber, Thomas Engstrøm, Steen Stender, Torben Hansen, Niels Grarup, Christina Ji-Young Lee, Charlotte Andersson, Christian Torp-Pedersen, Peter E. Weeke

**Affiliations:** 1 Unit of Clinical Biostatistics, Aalborg University Hospital, Aalborg, Denmark; 2 Department of Cardiology, Aalborg University Hospital, Aalborg, Denmark; 3 Faculty of Health and Medical Sciences, Novo Nordisk Foundation Center for Basic Metabolic Research, University of Copenhagen, Copenhagen, Denmark; 4 Department of Cardiology, Copenhagen University Hospital Gentofte, Hellerup, Denmark; 5 Department of Clinical Pharmacology, Bispebjerg and Frederiksberg Hospital, University of Copenhagen, Copenhagen, Denmark; 6 Department of Cardiology, Copenhagen University Hospital Rigshospitalet, Copenhagen, Denmark; 7 Department of Clinical Biochemistry, Copenhagen University Hospital Gentofte, Copenhagen, Denmark; 8 Department of Cardiology, Bispebjerg and Frederiksberg Hospital, Copenhagen, Denmark; Centro Cardiologico Monzino, ITALY

## Abstract

**Aims:**

Insulin resistance associates with development of metabolic syndrome and risk of cardiovascular disease. The link between insulin resistance and cardiovascular disease is complex and multifactorial. Confirming the genetic link between insulin resistance, type 2 diabetes, and coronary artery disease, as well as the extent of coronary artery disease, is important and may provide better risk stratification for patients at risk. We investigated whether a genetic risk score of 53 single nucleotide polymorphisms known to be associated with insulin resistance phenotypes was associated with diabetes and burden of coronary artery disease.

**Methods and results:**

We genotyped patients with a coronary angiography performed in the capital region of Denmark from 2010–2014 and constructed a genetic risk score of the 53 single nucleotide polymorphisms. Logistic regression using quartiles of the genetic risk score was performed to determine associations with diabetes and coronary artery disease. Associations with the extent of coronary artery disease, defined as one-, two- or three-vessel coronary artery disease, was determined by multinomial logistic regression.

We identified 4,963 patients, of which 17% had diabetes and 55% had significant coronary artery disease. Of the latter, 27%, 14% and 14% had one, two or three-vessel coronary artery disease, respectively. No significant increased risk of diabetes was identified comparing the highest genetic risk score quartile with the lowest. An increased risk of coronary artery disease was found for patients with the highest genetic risk score quartile in both unadjusted and adjusted analyses, OR 1.21 (95% CI: 1.03, 1.42, p = 0.02) and 1.25 (95% CI 1.06, 1.48, p<0.01), respectively. In the adjusted multinomial logistic regression, patients in the highest genetic risk score quartile were more likely to develop three-vessel coronary artery disease compared with patients in the lowest genetic risk score quartile, OR 1.41 (95% CI: 1.10, 1.82, p<0.01).

**Conclusions:**

Among patients referred for coronary angiography, only a strong genetic predisposition to insulin resistance was associated with risk of coronary artery disease and with a greater disease burden.

## Introduction

Insulin resistance is characterised by an impaired ability of the cells to use normal insulin concentrations and is associated with risk of developing type 2 diabetes and cardiovascular disease [1]. These relations are, however, complex and multifactorial. While the genetic underpinnings of type 2 diabetes and coronary artery disease are well characterised [2–4], limited knowledge on the genetic basis of insulin resistance is available. The latter notion is underlined by the fact that the majority of the loci identified in large genome-wide studies on type 2 diabetes are associated predominantly with insulin secretion and beta-cell function and not insulin resistance [2]. The lack of genetic loci identified owes in part to insulin resistance as a phenotype which is difficult to measure and is also influenced by lifestyle and environmental factors [5]. Given the coronary artery disease risk associated with insulin resistance, identifying patients genetically susceptible to develop insulin resistance is of considerable importance and may provide improved risk stratification for susceptibility to develop coronary artery disease.

In the largest study of insulin resistance to date by Lotta et. al. [6], genetic mechanisms of insulin resistance and associations with cardiometabolic disease traits were investigated in a population-based sample of 188,577 individuals. In this study, a triad of phenotypes as a surrogate of the golden standard measures of insulin resistance was investigated simultaneously. The phenotypes used to estimate insulin resistance were elevated fasting insulin levels (adjusted for body mass index [BMI]), decreased high-density lipoprotein (HDL) cholesterol, and elevated triglyceride levels. Elevated fasting insulin levels are a common measure of insulin resistance [7,8], and decreased HDL cholesterol and elevated triglyceride levels are also hallmarks in insulin resistant individuals [9]. In total, 53 single nucleotide polymorphisms (SNPs) were found to be associated with the aforementioned triad of phenotypes. Moreover, risk of developing type 2 diabetes and coronary artery disease in individuals with a genetic predisposition to insulin resistance was also established in the study by Lotta et al. [6]. Genome wide association studies (GWAS) have earlier identified SNPs associated with fasting insulin (adjusted for BMI). The majority of these SNPs also showed an association with lower HDL cholesterol and higher triglyceride levels, suggesting an influence on insulin resistance [9].

In the present study, independent of the study by Lotta et. al, we constructed a genetic risk score (GRS) based on 53 SNPs, known to be associated with insulin resistance phenotypes, in order to test if genetic predisposition to insulin resistance was associated with an increased risk of diabetes and coronary artery disease among patients susceptible for stable or unstable coronary artery disease. Furthermore, we aimed to test whether a genetic predisposition to insulin resistance was associated with severity of coronary artery disease, defined as one, two or three-vessel coronary artery disease.

## Methods

### Study cohort

The Copenhagen Cardiovascular Genetic study (COGEN) is comprised of approximately 80,000 patients admitted to six cardiology departments in the capital region of Copenhagen, Denmark, from 2010–2017. The present study is based on the first subset of COGEN participants that were genotyped, including patients with stable/unstable angina pectoris, non-ST elevation myocardial infarction (NSTEMI) or ST-elevation myocardial infarction (STEMI) who were referred to a coronary angiography (CAG) in the period 2010–2014. Baseline characteristics and results from the CAG were obtained from the Eastern Danish Heart Registry. The Eastern Danish Heart Registry covers data for all hospitals in Eastern Denmark where cardiac catheterisation and coronary revascularisations are performed. In this registry, CAG and percutaneous coronary interventions have been recorded since 1998 [10]. The registry includes information such as age, gender, smoking status, history of diabetes and hypertension, and indication for CAG, as well as the extent of vessel disease. We used the population-based cohort Inter99 for validating the GRS. The Inter99 study is a registered clinical trial (clinicaltrials.gov, NCT00289237), conducted by the Research Centre for Prevention and Health, Glostrup University Hospital, Glostrup, Denmark. It is a randomised non-pharmacological intervention study for prevention of ischemic heart disease. Of an age- and sex stratified random sample on 13,016 individuals from the Danish population in the south-western part of Copenhagen County, 6,784 individuals participated in baseline examinations. The study population was between 30 and 60 years old when entering the study in 1999 [11]. Detailed phenotypic characteristics of the Inter99 cohort have previously been published [12,13].

### Genotyping

For the present study, genotyping was performed using the Illumina Infinium Human CoreExome Beadchip-24v1.0 (Illumina, San Diego, CA, USA). Standard quality control was performed including, removal of variants with a minor allele frequency (MAF) less than 0.05 or variants that were out of Hardy-Weinberg equilibrium (p<0.0001). Moreover, it was ensured that all individuals had a genotyping rate > 5%. Variants were called using the Genome Reference Consortium Human Build 37 (GRCh37, hg19) as reference. In total, 539,004 variants in 5,671 individuals passed quality checks and were used for subsequent imputation. Imputation to the Haplotype Reference Consortium panel version 1.1 [14] was done on the Sanger Imputation Server, using Eagle for prephasing combined with positional Burrows–Wheeler transform for genotype imputation [15]. Patients that were not of European ancestry, based on principal components analysis, were excluded from the study population. See S1 File **Supplementary Material** for details on the genotyping, quality control and imputation.

The inter99 cohort was genotyped using the Cardio-Metabochip [16], and Human Exome BeadChip on an Illumina HiScan system (Illumina, San Diego, CA). Details have previously been described [17].

### Ethics

All data were de-identified prior to analyses. The ethics committee of Region North Jutland (N-20140048) approved the project and COGEN has permission from the Data Protection Agency (P-2019-202). The Inter99 study was approved by the Scientific Ethics Committee of the Capital Region of Denmark (KA98155), and from all Inter99 participants a written informed consent was obtained. The study was performed in accordance with the principles of the Declaration of Helsinki.

### Statistical analysis

Overall, 53 independent SNPs that were associated with each of the phenotypes fasting insulin levels (adjusted for BMI), lower HDL cholesterol level and higher triglyceride level in the integrative genomic approach analysis by Lotta et al. [6] were used to construct a weighted and an unweighted genetic risk score (GRS) (**S1 Table in S1 File**). In brief the GRSs were calculated as the sum of the risk alleles across all 53 SNPs. For the weighted score, the risk alleles were weighted by the effect found for the phenotype fasting insulin (adjusted for BMI) published in Lotta et al. [6]. This weighted score was normalised according to the respective weights, by dividing the score by the mean of the weights. Only results for the unweighted GRS will be presented in the following. Results for the weighted GRS can be found in **S1 File**.

Patient characteristics are presented as counts and proportions, median and inter quartile range, or by mean and standard deviation. For comparison of the GRSs in the Cogen and Inter99 cohorts a two sample t-test was used.

We performed multivariable logistic regression to evaluate associations of the GRSs with diabetes (history of diabetes: yes/no) and coronary artery disease, respectively. Patients were defined as having coronary artery disease, if they were registered with at least one-vessel coronary artery disease at the CAG. The GRSs were divided into quartiles (intervals (unweighted score): 1.Q: [38.8, 51.1], 2.Q: (51.1, 54], 3.Q: (54, 56.9], and 4.Q: (56.9, 68.3]) in order to evaluate the extent of the genetic burden (i.e. patients with the lowest level or genetic predisposition were in the first quartile [1.Q] and patients with the highest were in the fourth [4.Q]). Further analyses determining the extent of coronary artery disease were done by categorising the extent of coronary artery disease according to the presence of one -, two—and three-vessel coronary artery disease on the CAG. For this analysis multinomial logistic regression was performed, with the outcome being one -, two—or three-vessel coronary artery disease, compared with patients without coronary artery disease on their CAG. The reference group for the GRSs was for all analyses patients with the lowest genetic predisposition (i.e. 1.Q). Models were adjusted for age, gender, hypertension and smoking status. During sensitivity analyses, patients with prior history of acute myocardial infarction (AMI) were excluded from the study population, to ensure that the risk was not driven by patients with known coronary artery disease. We performed additional sensitivity analyses including the GRSs as continuous exposures in the multiple logistic regression models determining the risk of coronary artery disease, after visually inspecting a spline of the risk of coronary artery disease in relation to values of the GRSs. This was done to validate the results found in Lotta et. al. [6]. Furthermore the first two principal components from a principal component analysis identifying genetic ancestry on the final study population was added in the logistic regression models to ensure that risks were not affected by possible genetic ancestry remaining after exclusion of individuals of non-European ancestry. Effect modification from diabetes on the risk of coronary artery disease was explored by inclusion of a statistical interaction term in the logistic regression model. All statistical analyses were carried out in R version 3.4.0 [18]. A two-sided p value <0.05 was considered statistically significant.

## Results

### Patients characteristics

Overall, we identified 5,671 patients that were referred for CAG from 2010–2014 with stable/unstable angina pectoris, NSTEMI or STEMI. Of these, the final study population included in the present analysis was comprised of 4,963 patients after excluding individuals with a genetically determined non-European ancestry, and 122 patients with missing data on smoking status, hypertension, diabetes, or coronary artery disease. The mean age was 65.2 years (SD 11.3) and the majority were male (64.7%), non-smokers (75.7%), and had a history of hypertension (56.5%). A total of 17.8% had a history of diabetes. Stable angina pectoris was the main referral diagnosis for CAG (42.4%). Of patients referred for CAG, 55% had significant coronary artery disease, defined as one-vessel coronary artery disease (27%), two-vessel coronary artery disease (14%) or three-vessel coronary artery disease (14%) (**Table 1**). The mean unweighted insulin resistance GRS was 54.14 (SD: 4.27). In comparison, the mean unweighted insulin resistance GRS in the population-based Inter99 cohort was 53.97 (SD: 4.33) and no difference between the GRS in the two cohorts was found (p = 0.54). Boxplots comparing the GRSs in the Cogen and Inter99 cohort can be found in **S1 Fig in S1 File**. The Inter99 cohort available for genotyping in the present study was comprised of 6184 individuals. Characteristics of the cohort can be found in **S2 Table in S1 File.**

**Table 1:** Characteristics of the study population distributed on the quartiles (1.Q-4.Q) of the unweighted insulin resistance genetic risk score (IR GRS).

Variable	Level	1.Q (n = 1269)	2.Q (n = 1235)	3.Q (n = 1237)	4.Q (n = 1222)	Total (n = 4963)
IR GRS	Mean (SD)	48.6 (2.1)	52.7 (0.8)	55.4 (0.8)	59.4 (2.1)	54.0 (4.3)
Diabetes		229 (18.0)	219 (17.7)	199 (16.1)	237 (19.4)	884 (17.8)
Age (years)	Mean (SD)	65.5 (11.1)	65.1 (11.6)	65.3 (11.1)	65.1 (11.4)	65.2 (11.3)
Gender	Male	805 (63.4)	827 (67.0)	800 (64.7)	778 (63.7)	3210 (64.7)
Affected vessels	0	593 (46.7)	557 (45.1)	575 (46.5)	513 (42.0)	2238 (45.1)
	1	347 (27.3)	326 (26.4)	318 (25.7)	354 (29.0)	1345 (27.1)
	2	176 (13.9)	185 (15.0)	169 (13.7)	175 (14.3)	705 (14.2)
	3	153 (12.1)	167 (13.5)	175 (14.1)	180 (14.7)	675 (13.6)
BMI	Median [IQR]	27.2 [24.4, 30.5]	26.8 [24.2, 30.2]	26.5 [23.9, 30.1]	26.7 [23.9, 30.1]	26.8 [24.1, 30.2]
Smoking		320 (25.2)	282 (22.8)	312 (25.2)	291 (23.8)	1205 (24.3)
Hypertension		707 (55.7)	669 (54.2)	720 (58.2)	706 (57.8)	2802 (56.5)
Referral diagnosis to CAG	Angina pectoris	547 (43.4)	512 (41.5)	533 (43.3)	501 (41.2)	2093 (42.4)
	Chronic heart failure	67 (5.3)	68 (5.5)	67 (5.4)	80 (6.6)	282 (5.7)
	NSTEMI (AMI) ÷ Q-wave	110 (8.7)	126 (10.2)	109 (8.8)	133 (10.9)	478 (9.7)
	STEMI (AMI) ÷ Q-wave	232 (18.4)	216 (17.5)	228 (18.5)	193 (15.9)	869 (17.6)
	Unstable angina pectoris	84 (6.7)	105 (8.5)	94 (7.6)	86 (7.1)	369 (7.5)
	Other	219 (17.4)	206 (16.7)	201 (16.3)	222 (18.3)	848 (17.2)

AMI: Acute myocardial infarction. IQR: Interquartile range. Numbers are given as n (%), unless otherwise stated. BMI was not available for 42 of the patients, and the referral diagnosis was not available for 24 of the patients.

### Insulin resistance genetic risk score and risk of coronary artery disease and disease burden

**Fig 1:**
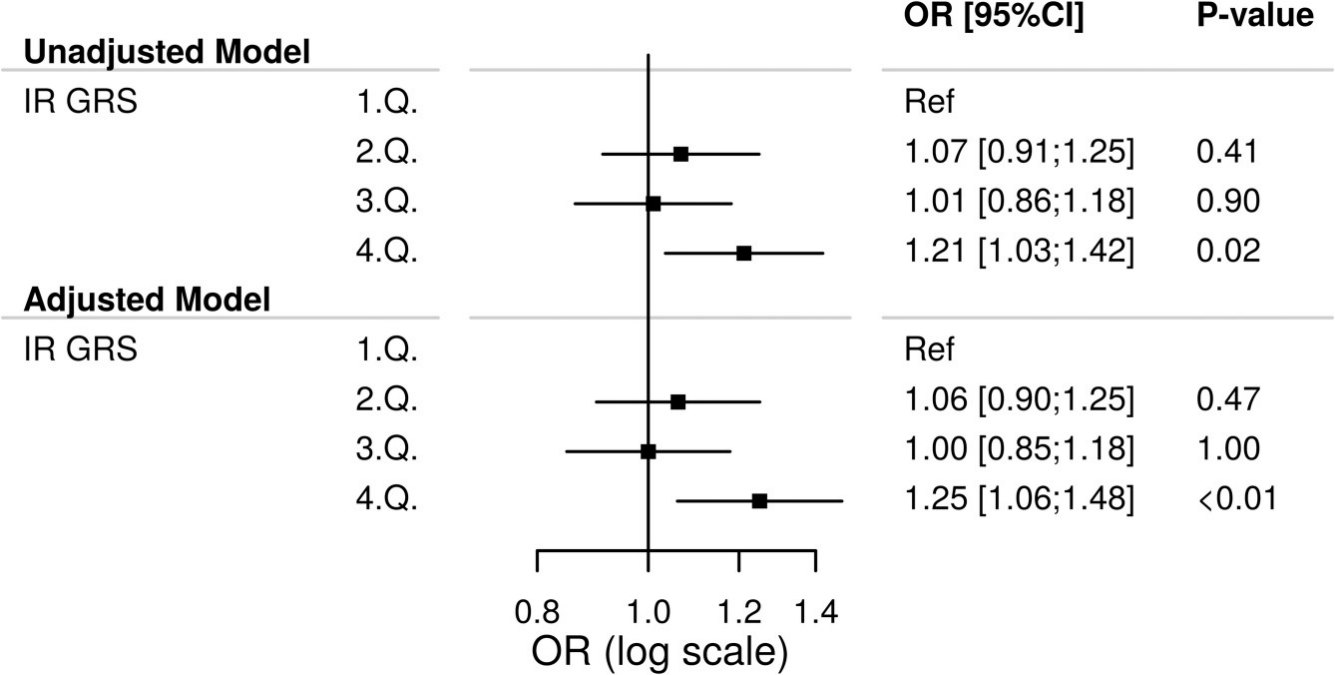
Association of the unweighted insulin resistance genetic risk score (IR GRS) with the risk of coronary artery disease among 4963 patients referred for coronary angiography. Logistic regression was used for this analysis. 1.Q,…,4.Q: 1. Quartile,…,4. Quartile. Ref: Reference. The Adjusted Model was adjusted for gender, age, smoking status and hypertension.

We tested the association between levels of the insulin resistance GRS with the risk of coronary artery disease, and identified patients within the highest GRS quartile (compared with patients within the lowest quartile) to be significantly associated with risk of coronary artery disease in both the unadjusted and adjusted analyses, OR (adjusted) 1.25 (95% CI: 1.06, 1.48, p<0.01, **Fig 1**). No significant association was identified for the second and third quartile (2.Q and 3.Q) of the GRS. Similar results were found for the weighted GRS (**S2 Fig in S1 File**).

**Fig 2:**
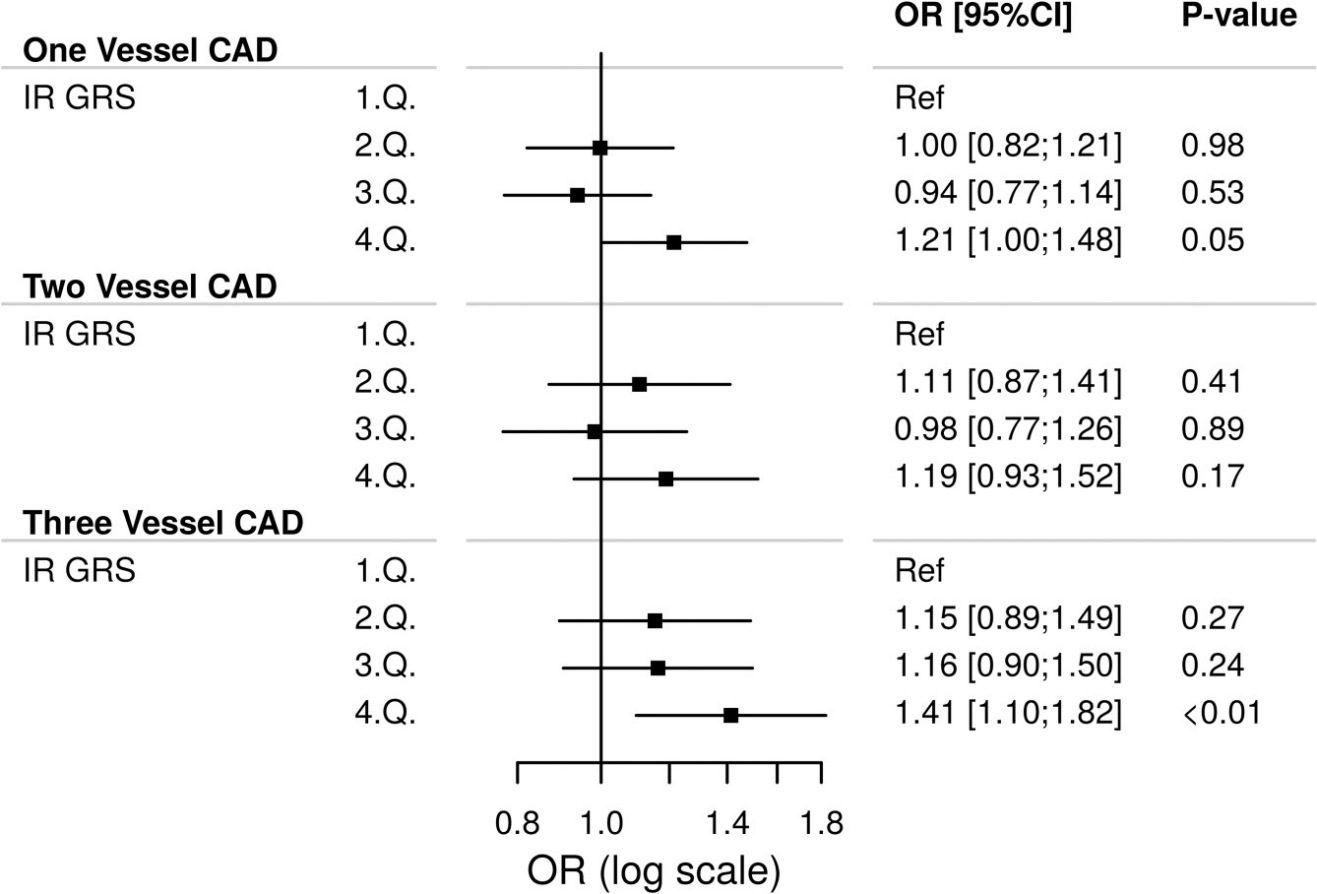
Risk of one, two or three-vessel coronary artery disease (CAD) by the unweighted insulin resistance genetic risk score (IR GRS) among 4963 patients referred for coronary angiography. A multinomial logistic regression model was used for this analysis. 1.Q,…,4.Q: 1. Quartile,…,4. Quartile. Ref: Reference. The model was adjusted for gender, age, smoking status and hypertension.

We also evaluated the extent of coronary artery disease based on the number of coronary vessels significantly affected by disease (i.e. one -, two—or three-vessel coronary artery disease) in relation to the insulin resistance GRS. A boxplot showing the distribution of the insulin resistance GRS according to number of coronary vessels affected can be found in **S3 Fig in S1 File**. A genetic predisposition to insulin resistance was associated with three-vessel coronary artery disease in the fourth quartile (4.Q) of the GRS by OR (adjusted) 1.41 (95% CI: 1.10, 1.82, p<0.01, **Fig 2**). The detailed models including estimates of confounders for both coronary artery disease and disease burden of coronary artery disease (only three-vessel coronary artery disease shown) can be found in **S4 and S5 Figs in S1 File,** respectively. Similar results were found for the weighted GRS (**S6 Fig in S1 File**) and for the sensitivity analyses excluding patients with prior AMI. No effect modification was found within the GRS among patients with and without history of diabetes. Likewise no remaining effect of genetic ancestry was found when adding the two principal components to the models. Sensitivity analyses including the GRS as continuous exposure (**S7-S9 Figs in S1 File**) showed a significant increased risk of coronary artery disease for the unweighted insulin resistance GRS at OR (adjusted) 1.01 (95% CI: 1.00, 1.03, p = 0.03).

### Insulin resistance genetic risk score and risk of diabetes

**Fig 3:**
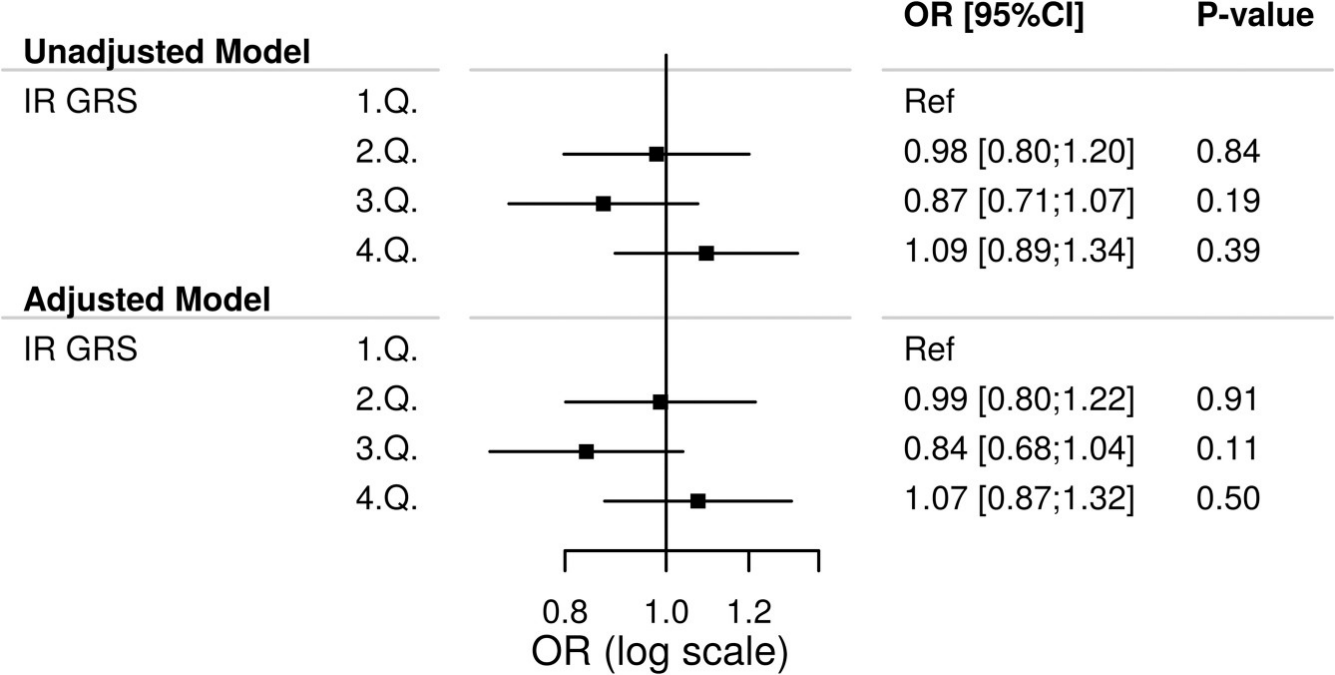
Association of the unweighted insulin resistance genetic risk score (IR GRS) with the risk of diabetes among 4963 patients referred for coronary angiography. Logistic regression was used for this analysis. 1.Q,…,4.Q: 1. Quartile,…,4. Quartile. Ref: Reference. The Adjusted Model was adjusted for gender, age, smoking status and hypertension.

We tested the association of the insulin resistance GRS with risk of diabetes using both the unweighted and weighted GRS. The unweighted GRS was not significantly associated with diabetes in both unadjusted and adjusted models (**Fig 3**). Detailed results including estimates of confounders can be found in **S10 Fig**. Similar results were found for the weighted GRS (**S11 Fig in S1 File**). A distribution of the unweighted and weighted GRS according to diabetes status can be found in the **S12 Fig in S1 File**.

## Discussion

The present study on the genetic association of insulin resistance on risk of diabetes, coronary artery disease and severity of coronary artery disease among 4,963 patients referred to CAG as part of standard care had three main findings. First, patients with a strong genetic predisposition to insulin resistance (i.e. patients in the fourth quartile (4.Q) of the constructed insulin resistance GRS) had increased risk of coronary artery disease. Second, the association between a strong genetic predisposition to insulin resistance and burden of coronary artery disease (i.e. one-, two- and three-vessel coronary artery disease) was most evident when looking at the risk of three-vessel coronary artery disease. No association between the insulin resistance GRS and one- or two-vessel coronary artery disease was identified. Third, no association between genetic predisposition to insulin resistance and diabetes was identified.

In the present study, we found a significant risk of three-vessel coronary artery disease for patients with the strongest genetic predisposition to insulin resistance compared to patients with the lowest genetic predisposition. Moreover, we observed a dose-response pattern of increasing risk of three-vessel coronary artery disease between the second, third and fourth quartile of the insulin resistance GRS compared to the first quartile, even though it was not significant for the second and third quartile. A less clear pattern was found for the risk of one- and two-vessel coronary artery disease. One limitation in the present study, that may have influenced study findings on the collective burden of coronary artery disease, is that information on all concurrent pharmacotherapy was not available. Moreover the cohort for the present study was highly selected, i.e. we did not have any healthy controls. One could speculate that because the patients are referred to the CAG due to some level of illness, they all might have a similar genetic risk of insulin resistance approximated by the GRS compared to a population-based cohort, which could make it difficult to differentiate between them. We computed the 53-SNP insulin resistance GRS also in the population-based Inter99 cohort, to determine whether this speculation holds. We found no significant difference in the GRSs when comparing the two cohorts, suggesting that the genetic risk of insulin resistance is not more pronounced in the Cogen cohort than in the Inter99 cohort. However three-vessel coronary artery disease represents the most severe form of coronary artery disease and is associated with worse long-term prognosis compared with less severe forms of coronary artery disease (i.e. one and two vessel coronary artery disease) [19]. We found that a high genetic risk of insulin resistance as probed by the 53 SNP GRS was associated with increased risk of significant three-vessel disease among patients referred for CAG. These findings are in line with the observation that patients with diabetes are susceptible to more extensive and dispersed coronary artery disease due to dyslipidemia and hyperinsulinemia [20]. Thus, patients with increased insulin resistance have an altered lipid metabolism which in turn augments atherosclerosis progression, endothelial dysfunction (e.g. generation of reactive oxygen species, the formation of advanced glycation end products, and systemic inflammation), plaque complications and medium and small vessels involvement [21,22]. Aside the abovementioned vasculopathy-associated risk factors for coronary artery disease development, patients with insulin resistance or diabetes are also more likely to be affected by other coronary artery disease related comorbid factors including obesity and hypertension. Thus, patients with diabetes experience worse outcomes following both percutaneous coronary interventions or surgery compared with patients without diabetes, and coronary artery disease remains the leading cause of death among patients with diabetes [23]. However, in the present study, the increased risk of three-vessel disease associated with a high genetic insulin resistance susceptibility was independent of known coronary artery disease risk factors including gender, diabetes, smoking, hypertension and obesity. While we did see a significant association between insulin resistance GRS and three-vessel coronary artery disease, no significant association was identified for insulin resistance GRS and one- and two vessel coronary artery disease. While more research into the underlying pathophysiology of single-vessel versus multi-vessel disease is needed, our findings support the notion that insulin resistance should be considered a systemic disorder affecting all vessels throughout the body, including coronary vessels.

Similar to previous findings by Lotta et. al. [6], we identified an association on the risk of coronary artery disease by the unweighted continuous 53-SNP insulin resistance GRS (OR (adjusted) 1.01 (95% CI: 1.00, 1.03, p = 0.03)). Moreover, our findings are also in agreement with the previous findings by Yaghootar et. al. where the risk of coronary artery disease associated with genetic insulin resistance predisposition demonstrated in a dose-response pattern: risk of coronary artery disease per allele of an 11-SNP insulin resistance GRS increased by OR 1.01 (95% CI: 1.01,1.02) [24]. Compared to previous studies, we also evaluated the extent of coronary artery disease, and found that a high genetic predisposition of insulin resistance seemed to be a more important factor in patients with more severe coronary artery disease. This might indicate that the genetics could help explain coronary artery disease in the most diseased group, and that it is more difficult to explain coronary artery disease in patients with less severe coronary artery disease based on a genetic predisposition of insulin resistance, as we did not find any significant associations for these groups. However, this finding needs further validation.

Genetically determined increased insulin resistance has previously been associated with risk of type 2 diabetes on several occasions. For example, Lotta et. al. found an increased risk of type 2 diabetes by OR 1.12 (95% CI 1.11, 1.14) per one standard deviation of the 53-SNP GRS [6], which was similar to the findings by Scott et. al. They found an increase in a 10-SNP insulin resistance GRS of the risk of type 2 diabetes with a Hazard Ratio at 1.08 (95% CI: 1.06,1.10) [25]. A similar result was found by Yaghootar et. al., where an 11-SNP insulin resistance GRS was significantly associated with an increased risk of type 2 diabetes at OR 1.04 (95% CI: 1.03,1.06) [24]. By contrast, we did not find a significant increase in the risk of diabetes in the present study. The lack of an association is likely due to the limited statistical power, considering the relatively small sample size used in the present study combined with the relatively small increment in type 2 diabetes risk previously identified. However, we did see a trend towards an increasing risk for high values of the GRS, although not significant. Another possibility of the lack of association with type 2 diabetes could be due to the fact that individuals with a high genetic predisposition of insulin resistance may not have been diagnosed with diabetes yet, and hence we were not able to identify them as having the disease. Thus, the risk of misclassification bias is a possibility, since patients with potential diabetes could have the CAG several years before the insulin resistance would result in diabetes. A better way to validate the insulin resistance genetic risk score, would be to associate it with the insulin resistance markers, fasting plasma glucose, HDL cholesterol and triglycerides, but these markers were not available for the study cohort.

## Conclusion and clinical implications

Among patients referred for CAG, only a strong genetic predisposition to insulin resistance (i.e. high genetic risk score burden) was associated with risk of coronary artery disease, and it was also associated with a greater disease burden. This pattern was most evident among patients with more severe coronary artery disease (i.e. three-vessel disease). Thus, improved patient risk stratification based on genetic insulin resistance predisposition may identify patients at risk of developing cardiovascular adverse events.

## Supporting information

S1 File Supplementary material. (PDF)
