# Supplementary material for: Insulin resistance genetic risk score and burden of coronary artery disease in patients referred for coronary angiography
Source: PLoS One. 2021 Jun 18;16(6):e0252855. doi: 10.1371/journal.pone.0252855 (PMC8213191; doi:10.1371/journal.pone.0252855)
Supplement: S1 File — (PDF) [file pone.0252855.s001.pdf]

Supplementary Material

Genotyping, quality control and imputation

For genotyping the Ilumina HiScan system was used, at the laboratory of the Novo Nordisk Foundation Centre for Basic Metabolic Research, at Symbion, Copenhagen, Denmark.

The standard pipeline in Illumina Genome Studio software was applied for genotype calling. SNPs were updated to build 37 (i.e. hg19). A total of 5904 individuals and 547644 SNPs were included before quality control (QC). A total of 5671 individuals and 539004 SNPs remained after QC for further analysis. The following criteria were used for QC:

1) A call-rate below 95% (removed n=46 individuals)
2) Extreme positive or negative inbreeding coefficients (removed n=56 individuals)
3) Outliers by ethnicity using principal component analysis (PCA) (removed n=566 individuals)
4) Unknown first degree relative relations found by identical descent (IBD) analysis where only the relative with the highest call-rate for each pedigree-pair was retained (removed n=63 individuals)
5) Duplicated samples (excluded n=16 individuals)
6) Disagreement of sex between genotype and phenotype data (removed n=29 individuals)

The software used for QC was a combination of PLINK, Python and R.

Imputation was done on the Sanger Imputation server on January 5, 2017. A VCF file was prepared from genotypes from the Ilumina Core Exome chip for the purpose, and it was aligned to the forward strand of GRCh37 according to instructions from the Sanger Imputation service. The following exclusions were made prior to imputation:

1) Non-autosomal variants
2) Variants with more than 5% missing calls
3) Variants with a minor allele frequency less than 0.05
4) Variants that diverged from Hardy-Weinberg equilibrium with a p-value at most 0.0001

After exclusions the total amount of variants genotyped was 260792 in 5671 individuals. These were prephased using Eagle and imputed using positional Burrows–Wheeler transform (PBWT) to the Haplotype Reference Consortium (r1.1).

53 SNP's associated with Insulin Resistance

SNP	Chr	Pos	Locus	Effect Allele	Other Allele	Effect	Genotyped
rs4846565	1	219722104	RNU5F-1/LYPLAL1	G	A	0,022	Imputed
rs683135	1	39895460	MACF1	A	G	0,014	Imputed
rs17386142	1	50815783	DMRTA2	C	T	0,024	Imputed
rs11577194	1	110500175	CSF1	T	C	0,011	Imputed
rs9425291	1	172312769	DNM3	A	G	0,015	Genotyped
rs10195252	2	165513091	COBLL1/GRB14	T	C	0,029	Genotyped
rs2943645	2	227099180	IRS1	T	C	0,032	Imputed
rs2249105	2	65287896	CEP68	A	G	0,016	Genotyped
rs492400	2	219349752	USP37	T	C	0,01	Imputed
rs308971	3	12116620	SYN2/PPARG	G	A	0,036	Genotyped
rs3864041	3	15185634	COL6A4P1	T	C	0,011	Imputed
rs295449	3	47375955	KLHL18	A	G	0,011	Imputed
rs11130329	3	52896855	TMEM110-MUSTN1	A	C	0,024	Genotyped
rs9881942	3	123082416	ADCY5	A	G	0,013	Genotyped
rs645040	3	135926622	MSL2	T	G	0,014	Genotyped
rs3822072	4	89741269	FAM13A	A	G	0,02	Imputed
rs6822892	4	157734675	PDGFC	A	G	0,024	Genotyped
rs2699429	4	3480136	DOK7	C	T	0,011	Imputed
rs4865796	5	53272664	ARL15/FST	A	G	0,025	Imputed
rs459193	5	55806751	ANKRD55	G	A	0,025	Genotyped
rs4976033	5	67714246	PIK3R1	G	A	0,015	Genotyped
rs6887914	5	112711486	MCC	C	T	0,013	Imputed
rs1045241	5	118729286	TNFAIP8	C	T	0,012	Imputed
rs2434612	5	158022041	EBF1	G	A	0,016	Imputed
rs966544	5	173350405	CPEB4	G	A	0,012	Imputed
rs2745353	6	127452935	RSPO3	T	C	0,019	Imputed
rs12525532	6	35004819	ANKS1A	T	C	0,019	Imputed
rs6937438	6	43815364	LOC100132354	A	G	0,013	Imputed
rs9492443	6	130398731	L3MBTL3	C	T	0,014	Imputed
rs3861397	6	139828916	LOC645434	G	A	0,014	Imputed
rs17169104	7	15883727	MEOX2	G	C	0,02	Imputed
rs972283	7	130466854	KLF14	G	A	0,022	Genotyped
rs2126259	8	9185146	PPP1R3B	T	C	0,041	Genotyped
rs1011685	8	19830769	LPL	C	T	0,019	Imputed
rs4738141	8	72469742	EYA1	G	A	0,014	Genotyped
rs7005992	8	126528955	TRIB1	C	G	0,016	Imputed
rs498313	9	78034169	MIR548H3	A	G	0,013	Imputed
rs10995441	10	64869239	NRBF2	G	T	0,014	Imputed
rs11231693	11	63862612	MACROD1	A	G	0,036	Imputed
rs17402950	12	14571671	ATF7IP	G	A	0,027	Imputed
rs718314	12	26453283	ITPR2	G	A	0,017	Genotyped
rs7973683	12	124449223	CCDC92/DNAH10	C	A	0,019	Imputed
rs7323406	13	111628195	ANKRD10	A	G	0,015	Imputed

rs7176058	15	39464167	C15orf54	A	G	0,013	Imputed
rs8032586	15	73081067	LOC100287559	C	T	0,019	Imputed
rs754814	17	4657034	ZMYND15	T	C	0,011	Genotyped
rs7227237	18	47174679	LIPG	C	T	0,017	Genotyped
rs731839	19	33899065	PEPD	G	A	0,025	Genotyped
rs8101064	19	7293119	INSR	T	C	0,042	Imputed
rs4804833	19	7970635	MAP2K7	A	G	0,016	Genotyped
rs4804311	19	8615589	MYO1F	A	G	0,019	Imputed
rs6066149	20	45602638	EYA2	G	A	0,013	Imputed
rs132985	22	38563471	PLA2G6	C	T	0,016	Genotyped

Table 1. *SNP's and effect sizes as reported in Lotta et al. [6].*

Comparison of the 53 SNP Insulin Resistance Genetic Risk Score in the Cogen and Inter99 cohorts

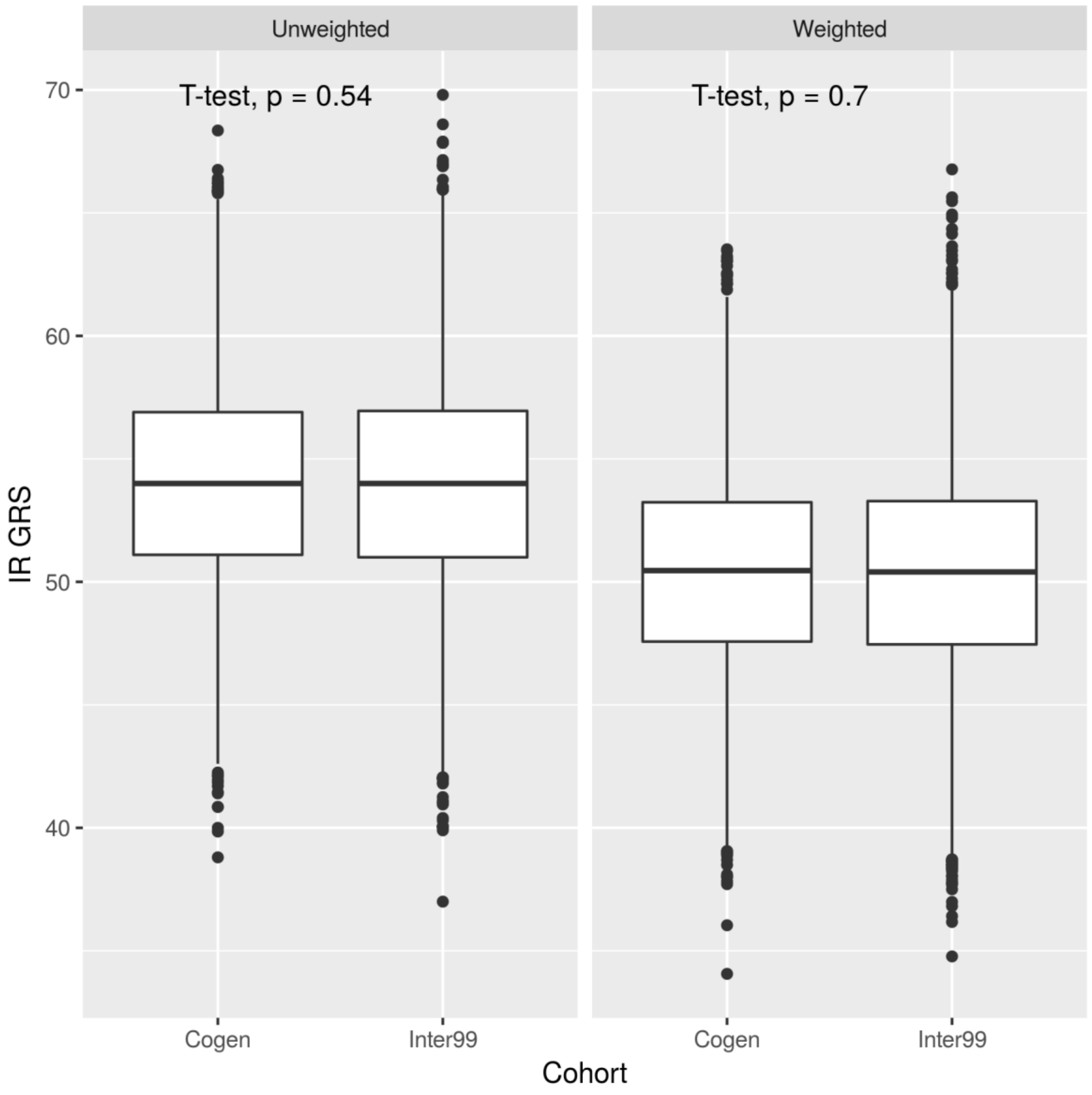

Fig 1. *Distribution of the unweighted and weighted Insulin Resistance Genetic Risk Score (IR GRS) for the Cogen and Inter99 cohorts.*

The Inter99 Cohort

Variable	Level	Total
Age	mean (sd)	46.23 (7.93)
Sex	male	3,019 (48.82)
BMI	median [iqr]	25.61 [23.14, 28.63]
	missing	5
IR GRS	mean (sd)	53.97 (4.33)
Smoking		3,986 (64.77)
	missing	30
Hypertension		1,343 (21.72)
Diabetes		314 (6.35)
	missing	1237
Stroke		227 (3.67)
CAD		385 (6.23)
AMI		132 (2.13)

Table 2. Characteristics of the Inter99 cohort. IR GRS: Insulin Resistance Genetic Risk Score. AMI: Acute Myocardial Infarction. BMI: Body Mass Index.

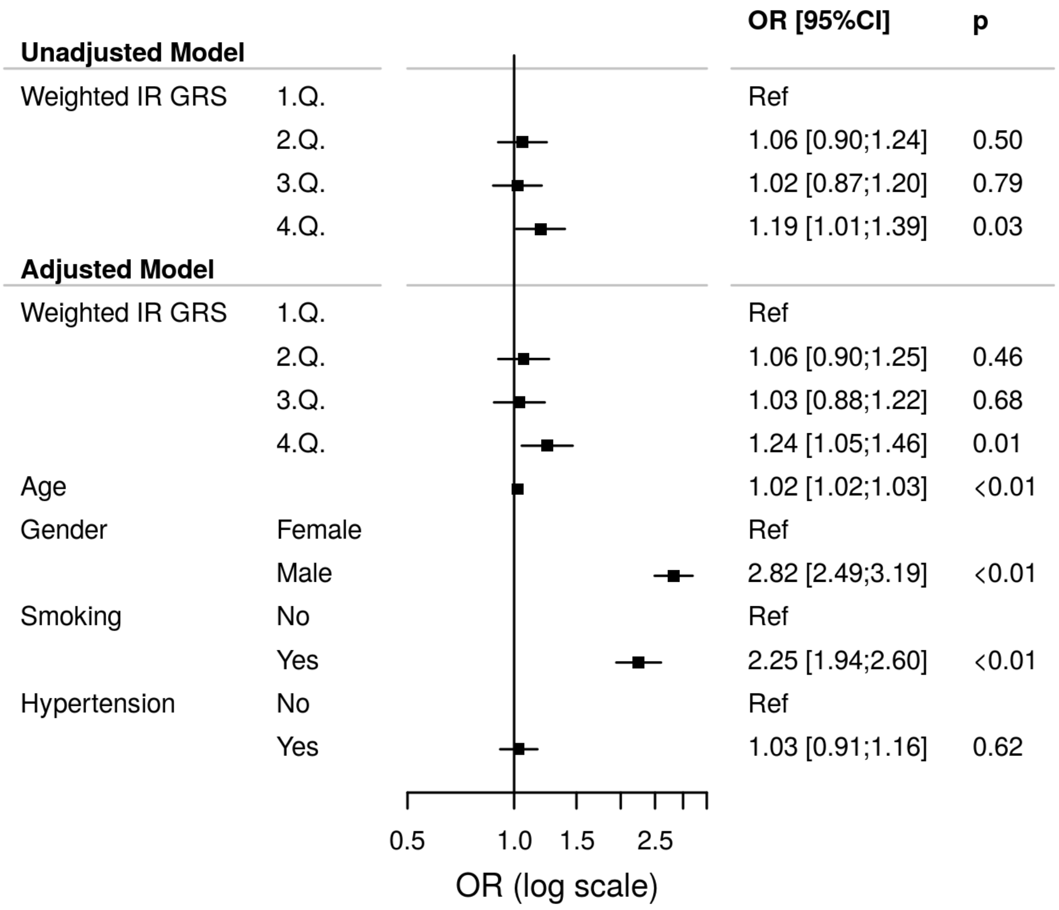

Fig 2. *Association of the weighted Insulin Resistance Genetic Risk Score (IR GRS) with the risk of coronary artery disease (binary outcome). Ref: Reference. p: p-value.*

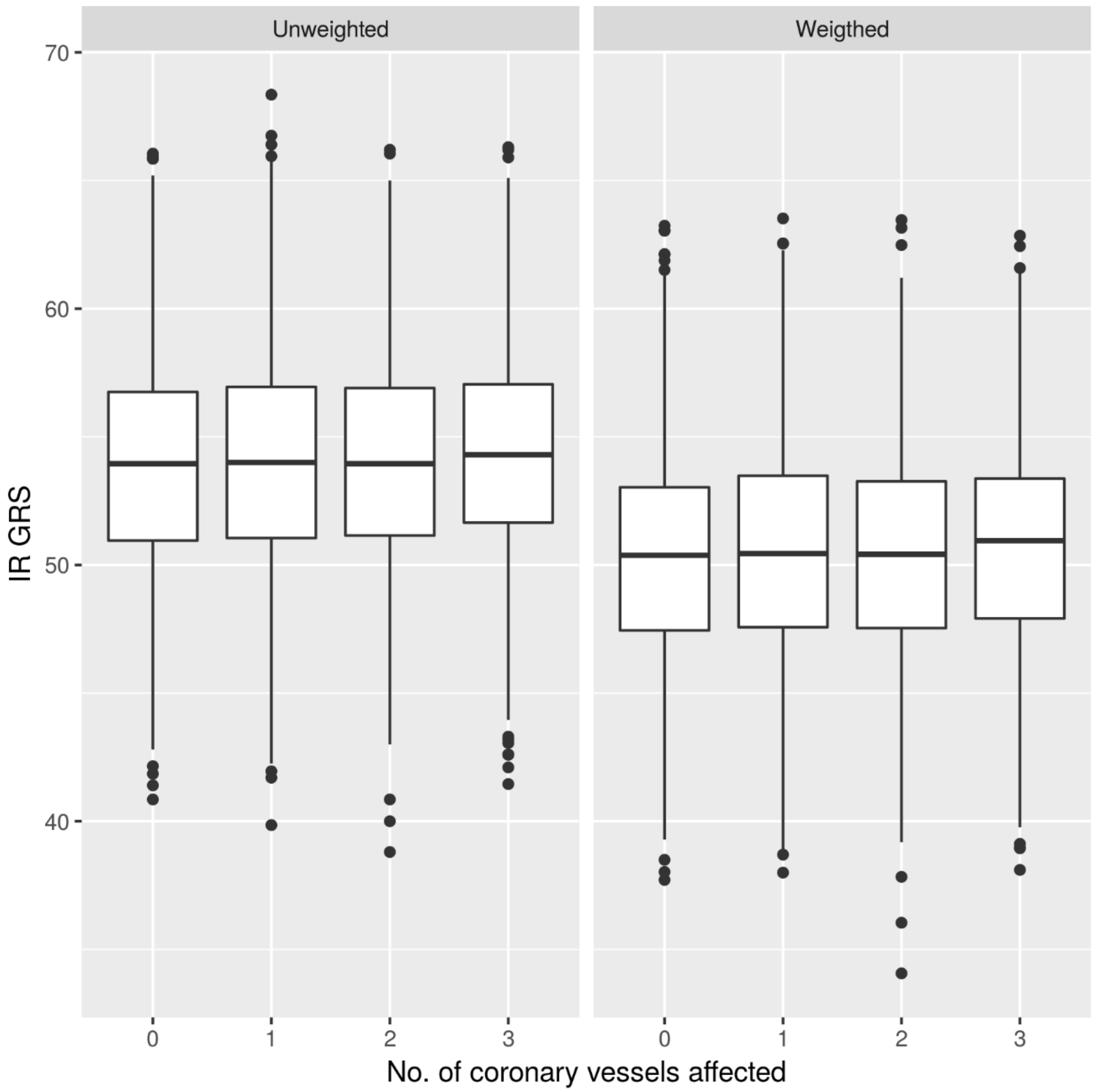

Fig 3. *Distribution of the standardized Insulin Resistance Genetic Risk Score (IR GRS) by the number of affected coronary vessels.*

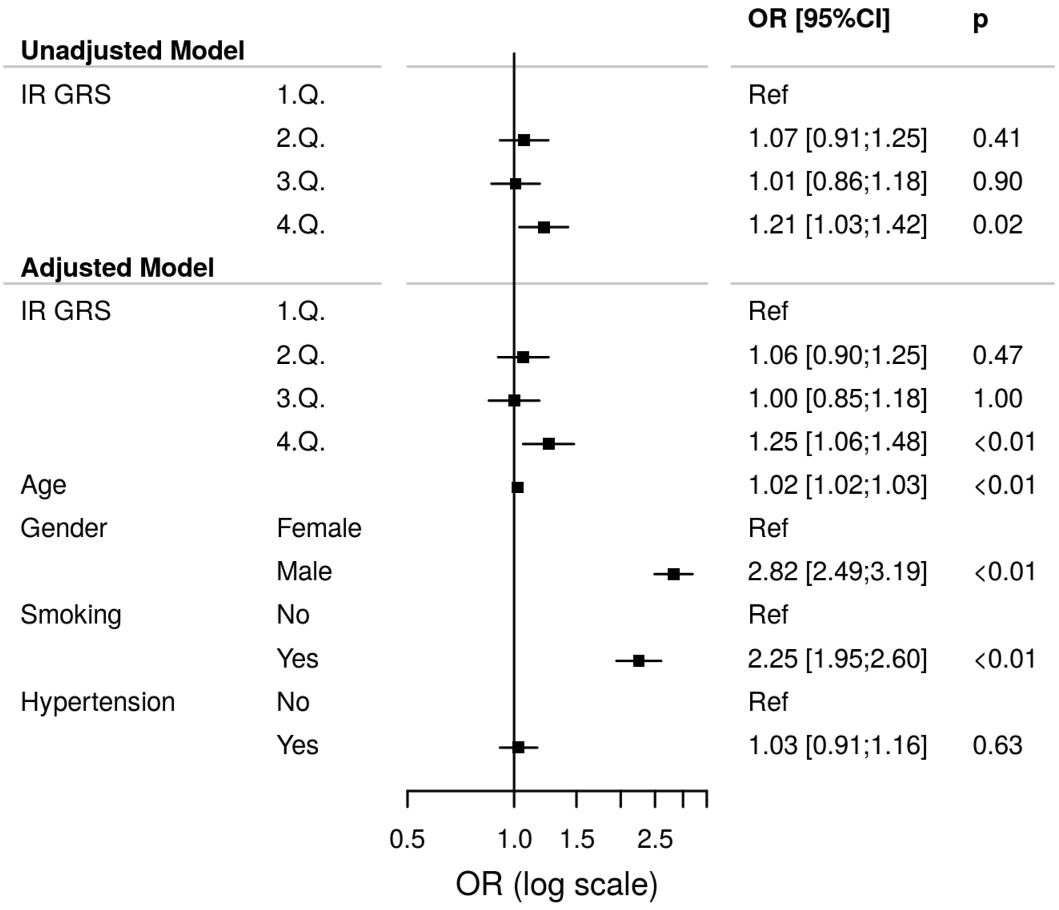

Fig 4. *Association of the unweighted Insulin Resistance Genetic Risk Score (IR GRS) with the risk of coronary artery disease (binary outcome). Ref.: Reference. p: p-value.*

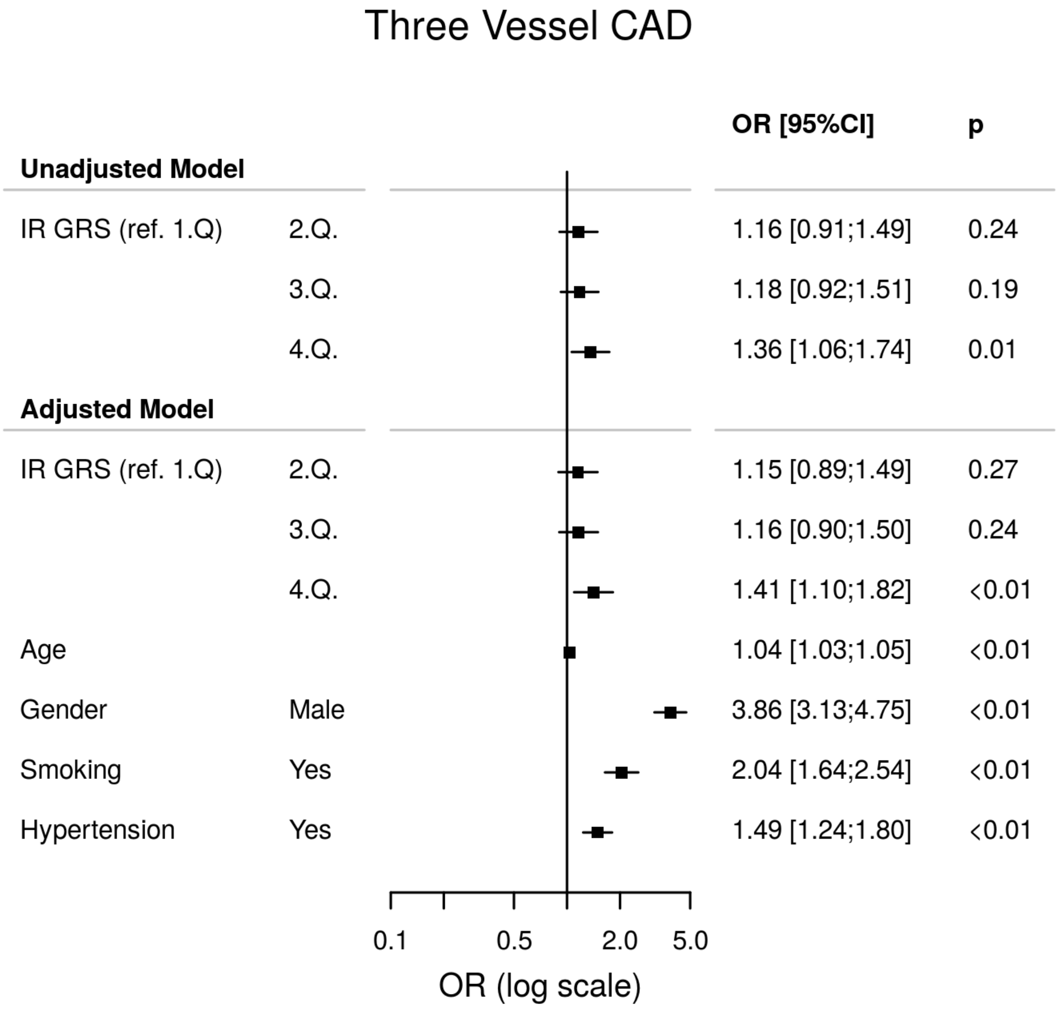

Fig 5. *Risk of three vessel coronary artery disease by the unweighted Insulin Resistance Genetic Risk Score (IR GRS). Ref.: Reference. p: p-value.*

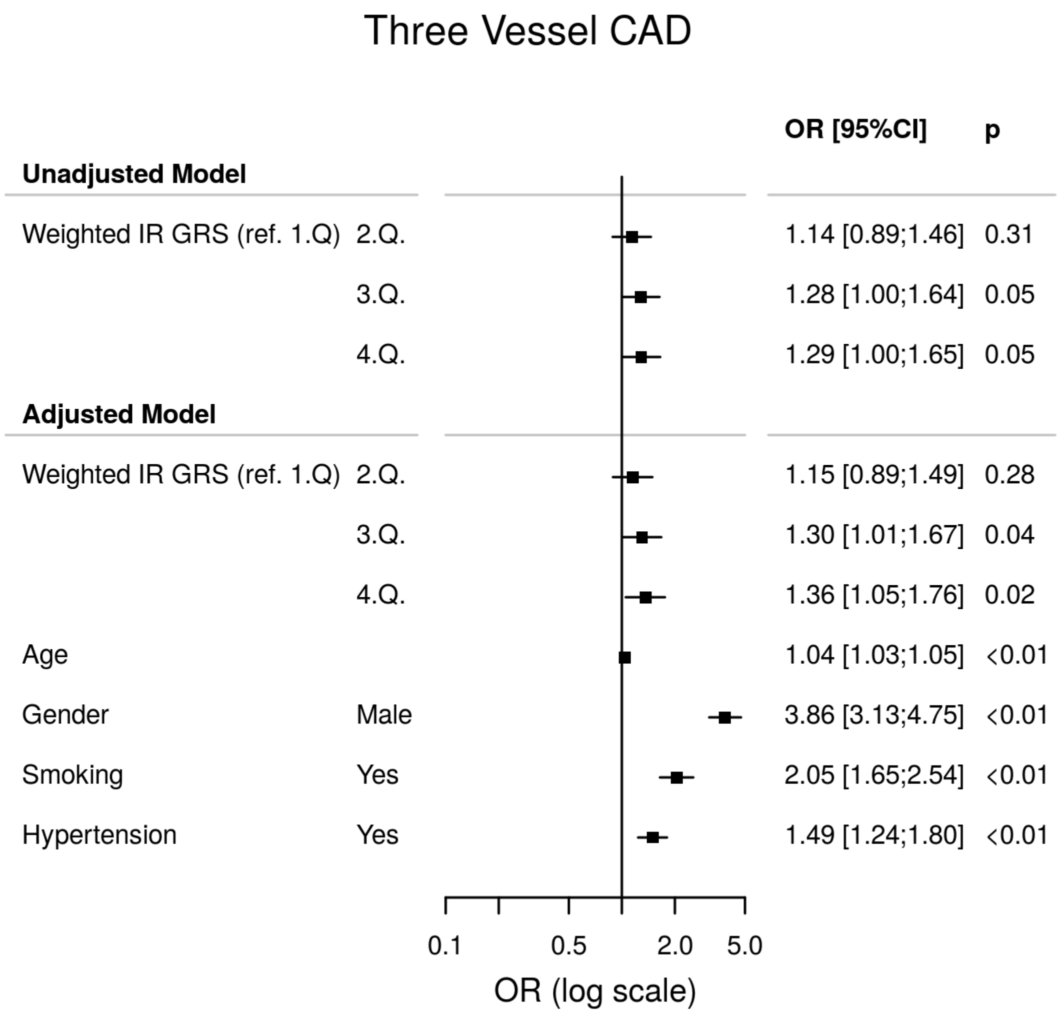

Fig 6. *Risk of three vessel coronary artery disease by the weighted Insulin Resistance Genetic Risk Score (IR GRS). Ref.: Reference. p: p-value.*

Including the GRS as a continuous exposure

Spline of the standardized GRS in relation to odds of coronary artery disease

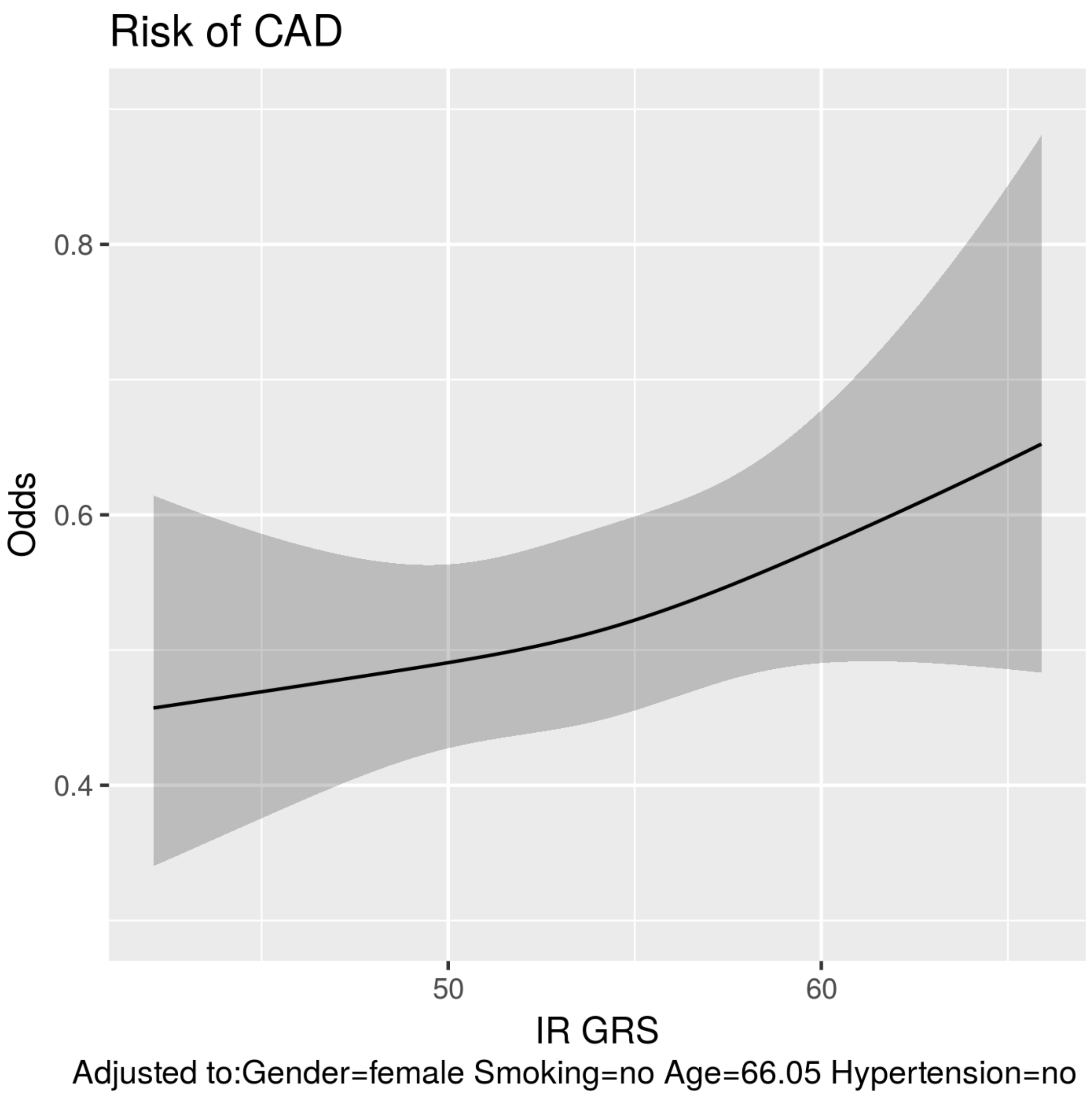

Fig 7. *Risk of coronary artery disease (in odds), according to level of the unweighted IR GRS. The baseline is according to a non-smoking female, who is around 66 years old and does not have hypertension.*

Association of The Insulin Resistance GRS with the binary outcome coronary artery disease

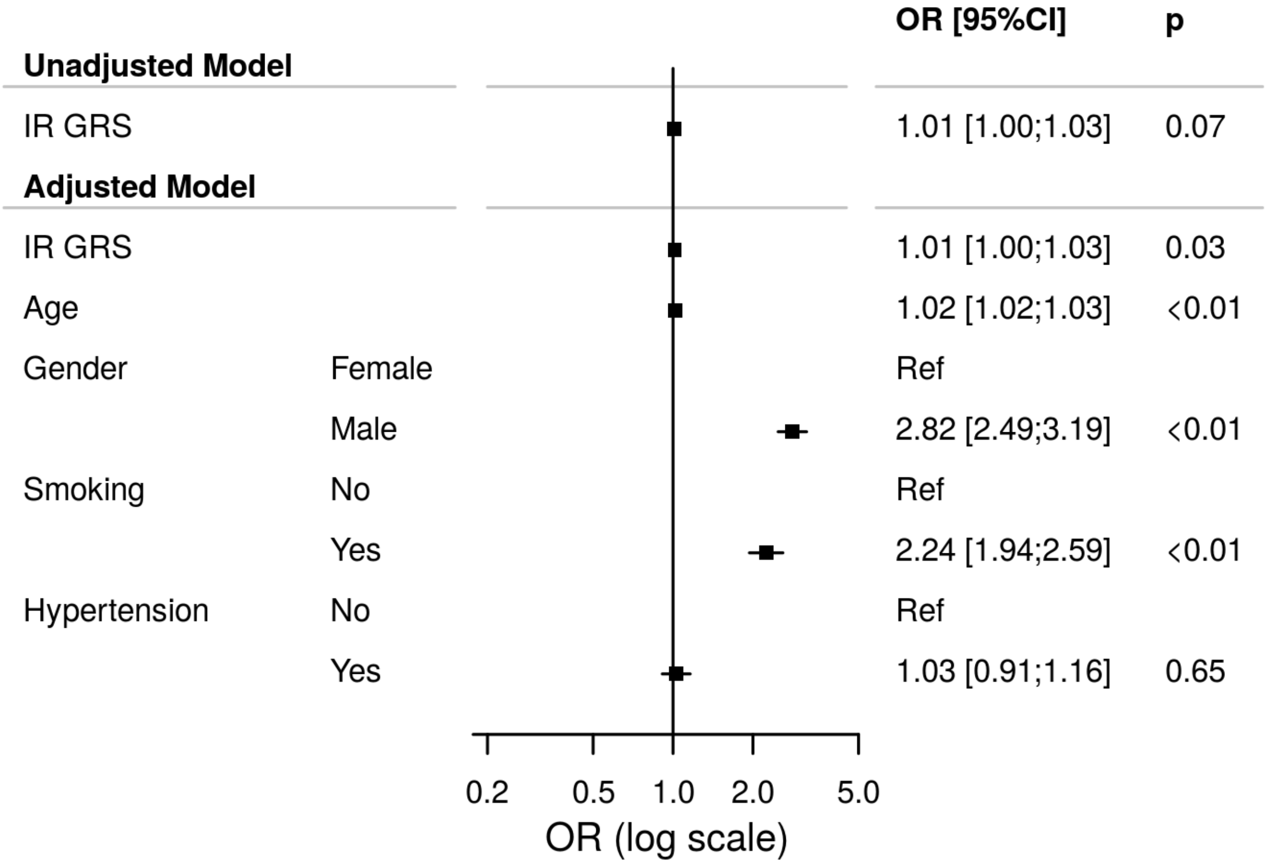

Fig 8. *Risk of coronary artery disease by the continuous unweighted Insulin Resistance Genetic Risk Score (IR GRS). Ref: Reference. p: p-value.*

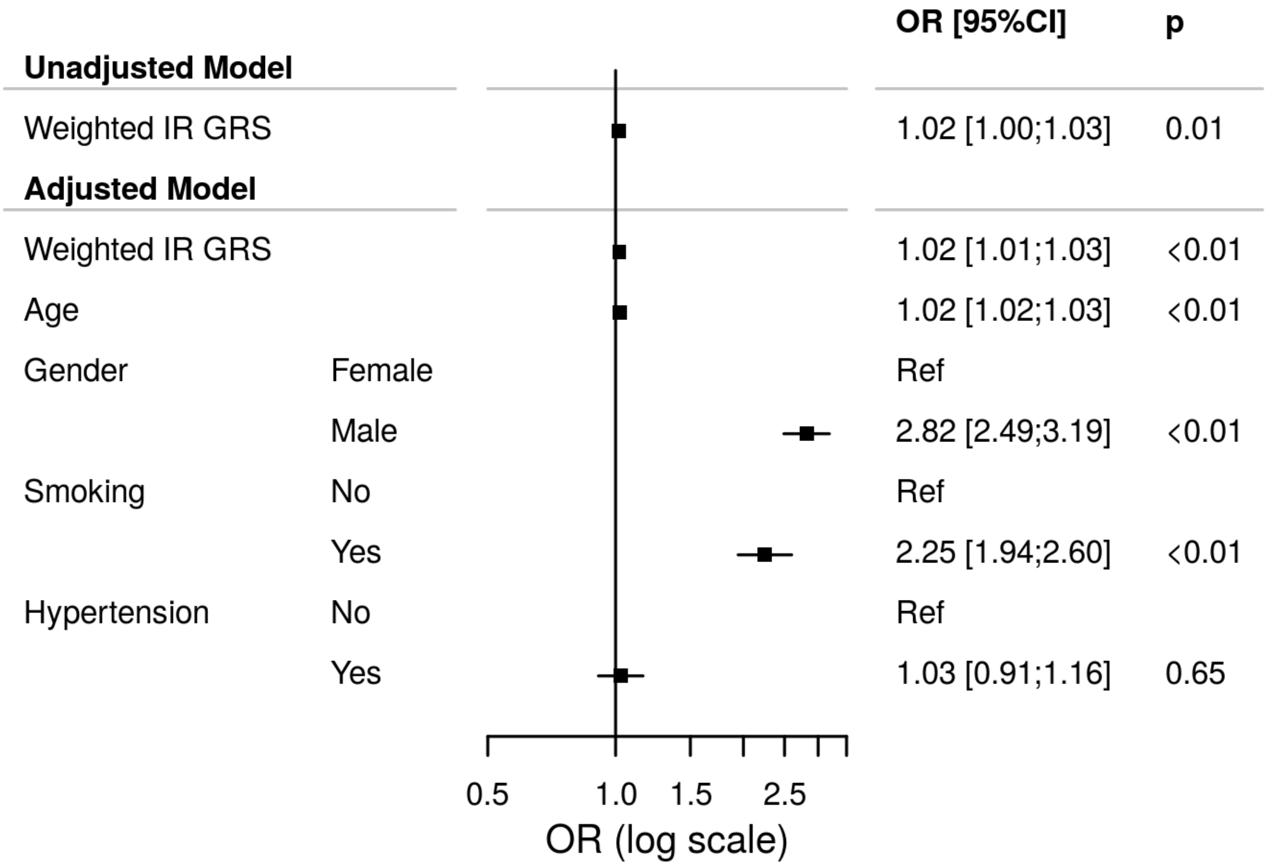

Fig 9. *Risk of coronary artery disease by the continuous weighted Insulin Resistance Genetic Risk Score (IR GRS). Ref: Reference. p: p-value.*

Association of The Insulin Resistance GRS with diabetes

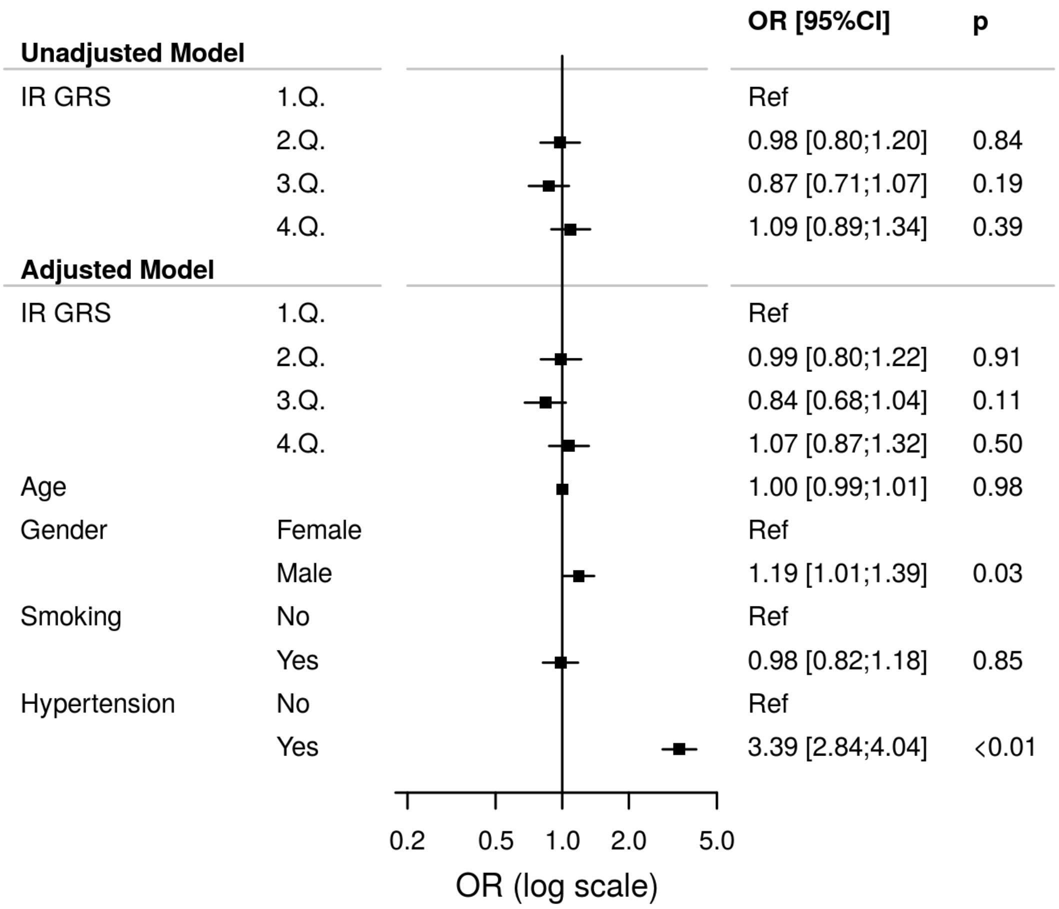

Fig 10. *Association of the unweighted Insulin Resistance Genetic Risk Score (IR GRS) with the risk of diabetes. Ref.: Reference. p: p-value.*

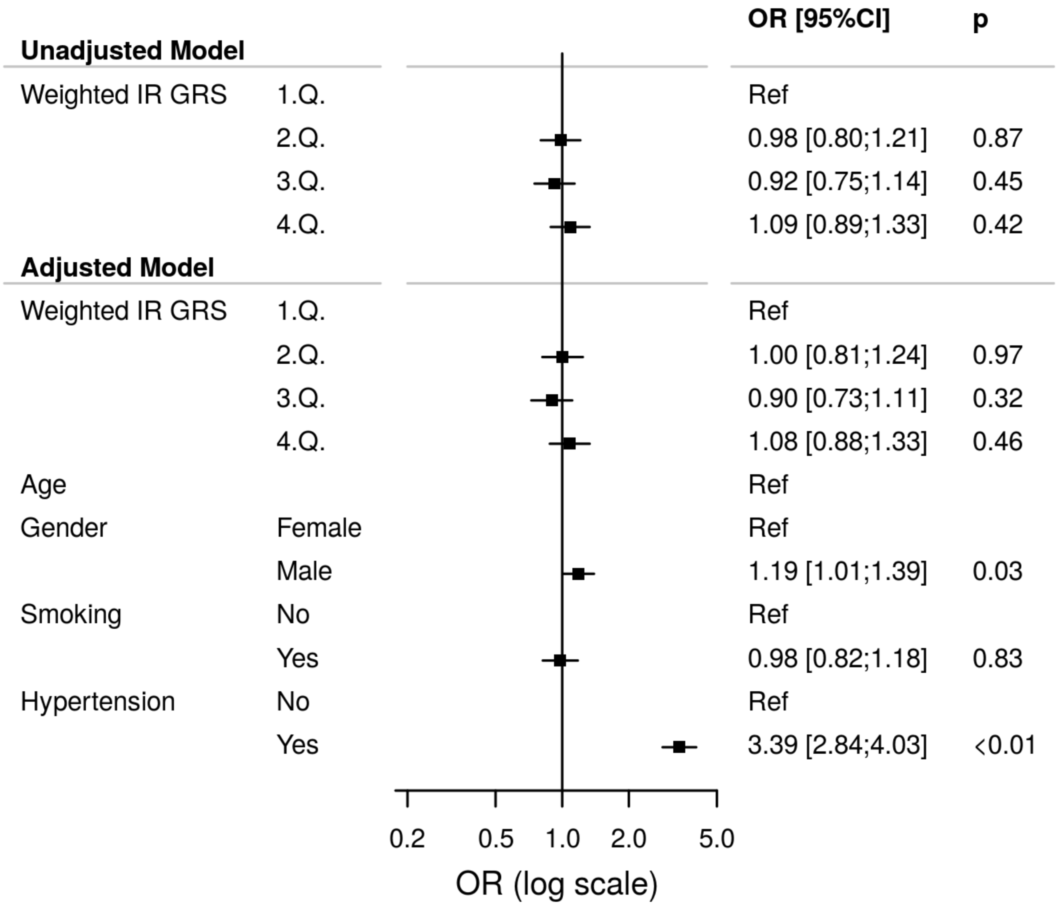

Fig 11. *Association of the weighted Insulin Resistance Genetic Risk Score (IR GRS) with the risk of diabetes. Ref.: Reference. p: p-value.*

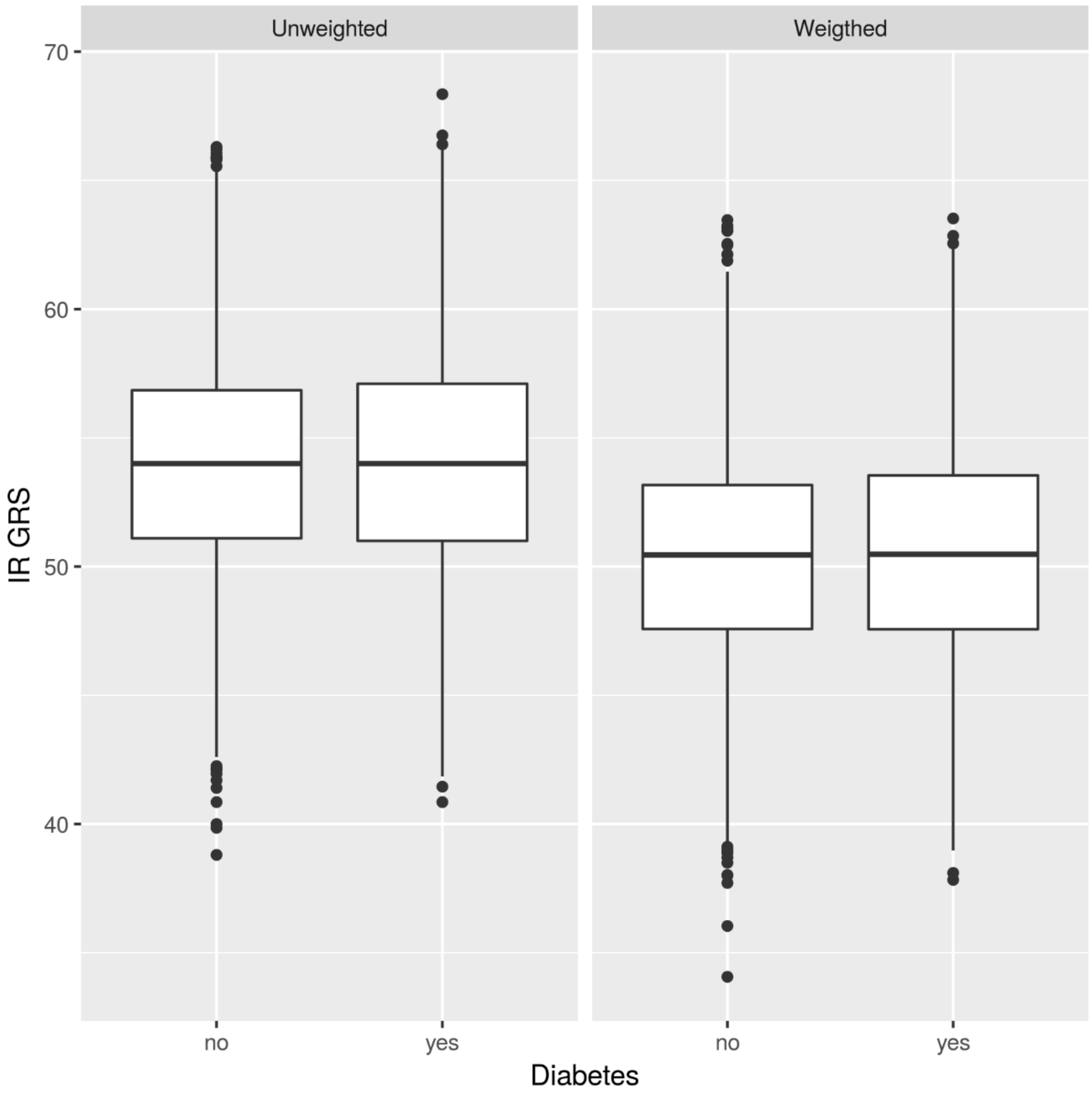

Fig 12. *Distribution of the Insulin Resistance Genetic Risk Score (IR GRS) by diabetes.*
